# Quality of life, immunomodulation and safety of adjuvant mistletoe treatment in patients with gastric carcinoma – a randomized, controlled pilot study

**DOI:** 10.1186/1472-6882-12-172

**Published:** 2012-10-03

**Authors:** Kab-Choong Kim, Jeong-Hwan Yook, Jürgen Eisenbraun, Byung-Sik Kim, Roman Huber

**Affiliations:** 1 Division of Stomach surgery, Department of Surgery, Asan Medical Center, College of Medicine, University of Ulsan, Seoul, Korea; 2 Abnoba GmbH, Pforzheim, Germany; 3 Center for Complementary Medicine, University Medical Center Freiburg, Freiburg, Germany

**Keywords:** Qol, EORTC QLQ-C30, QLQ-STO22, 5-FU, Viscum album

## Abstract

**Background:**

Mistletoe (Viscum album L.) extracts are widely used in complementary cancer therapy. Aim of this study was to evaluate safety and efficacy of a standardized mistletoe extract (abnobaVISCUM® Quercus, aVQ) in patients with gastric cancer.

**Patients and Methods:**

32 operated gastric cancer patients (stage Ib or II) who were waiting for oral chemotherapy with the 5-FU prodrug doxifluridine were randomized 1:1 to receive additional therapy with aVQ or no additional therapy. aVQ was injected subcutaneously three times per week from postoperative day 7 to week 24 in increasing doses. EORTC QLQ-C30 and -STO22 Quality of Life questionnaire, differential blood count, liver function tests, various cytokine levels (tumor necrosis factor (TNF)-alpha, interleukin (IL)-2), CD 16^+^/CD56^+^ and CD 19^+^ lymphocytes were analyzed at baseline and 8, 16 and 24 weeks later.

**Results:**

Global health status (p <0.01), leukocyte- and eosinophil counts (p ≤0.01) increased significantly in the treatment group compared to the control group. Diarrhea was less frequently reported (7% vs. 50%, p=0.014) in the intervention group. There was no significant treatment effect on levels of TNF-alpha, IL-2, CD16^+^/CD56^+^ and CD 19^+^ lymphocytes and liver function tests measured by ANOVA.

**Conclusion:**

Additional treatment with aVQ is safe and was associated with improved QoL of gastric cancer patients. ClinicalTrials.Gov Registration number NCT01401075.

## Background

Gastric cancer is the most frequent cancer in Korea and the second most common cancer worldwide [1,2] Surgical resection remains the primary curative treatment option with overall 5-year survival rates of 15% to 35% [3]. The survival rate for patients with gastric cancer has only slightly be improved in the last years by using technical advances in surgery and adjuvant chemotherapy [4].

It is well known that surgical stress suppresses the immune system. Granulocyte function, numbers of natural killer (NK) cells and T-helper lymphocytes decrease after major surgery [5,6]. Furthermore, chemotherapeutics like 5-FU frequently (≥1:100, <1:10) cause immunosuppression (neutropenia, myelosupression) with increased risk of infection [7] and may have a negative impact on patients´quality of life [8]. Adjuvant 5-FU as monotherapy or in combination with other chemotherapies has been effective in gastric cancer patients UICC stage I-IV to reduce mortality [9] and is a standard therapy in patients with gastric cancer stage II-III [10]. Instead of 5-FU in Japan and Korea also the 5-FU prodrug doxifluridine (5-DFUR), which can be applied orally, is used [11].

Aqueous mistletoe extracts (Viscum album L.) have been widely used in complementary cancer therapy for decades [12]. Mistletoe extracts as well as isolated mistletoe-lectins were shown to have immunomodulatory properties by enhancing the secretion of cytokines and the number and activity of immunological effector cells like NK-cells [13,14] and T-lymphocytes [15]. NK-cell activity can predict the prognosis of patients with gastric cancer [16] and NK-cells are reduced stage dependently in Korean gastric cancer patients [17] but there are not many studies on this issue. NK-cells were, therefore, chosen as an immunological parameter in this trial. Low Interleukin (IL)-2 is regarded as disadvantageous for sufficient T-cell response [18] and was, therefore, also included into the analysis.

Moreover, mistletoe extracts inhibited tumor cell proliferation and tumor growth in numerous *in vitro* studies and animal experiments [19-22]. The effects could be attributed to the specific components mistletoe lectin I–III. [22,23]. abnobaVISCUM® Q (aVQ) is one of the mistletoe preparations with the highest content of mistletoe lectins on the market [24].

It has been shown in clinical trials, especially with breast or colorectal cancer patients, that QoL improved under mistletoe therapy [12,25]. In operated patients with gastric cancer during chemotherapy, mistletoe treatment has not yet been investigated [25]. We, therefore, conducted a randomized clinical trial to investigate the effect on quality of life, immunomodulation and safety of adjuvant subcutaneous mistletoe treatment on patients with gastric cancer receiving chemotherapy after operation.

## Methods

### Patients

32 patients, sixteen each for the treatment group and for the control group, were recruited for this pilot trial from March 2006 to April 2008. Inclusion criteria were: postoperative gastric cancer (stage Ib or II) waiting for oral chemotherapy with the orally applied 5-FU prodrug and intermediate metabolite of capecitabine doxifluridine (5-DFUR), aged between 19 and 70 years, Eastern Cooperative Oncology Group (ECOG) performance status 0 or 1, normal liver function and renal function. Exclusion criteria were: inability to answer the QoL scales, concomitant therapy with steroids or biological response modifiers, individual hypersensitivity to mistletoe preparations, pregnancy or lactating and participation in another clinical trial.

**Figure 1:**
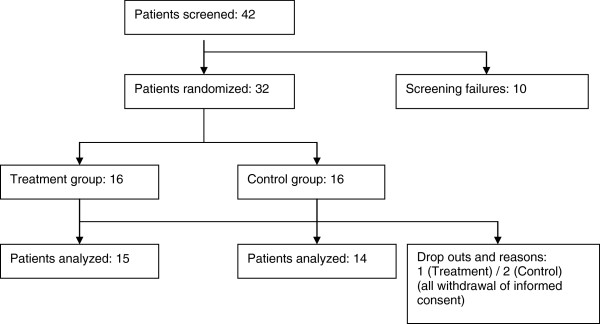
Flow of the study.

The protocol was approved by the Institutional Review Board of ASAN Medical Center (irbreview@amc.seoul.kr). Written informed consent was obtained from all patients. The flow of the study is shown in Figure 1.

### Study design

The study was prospective, controlled and randomized, comparing two arms. Patients were allocated on the basis of unstratified block randomization (block size: 4) to the intervention group (aVQ) or no additional therapy (control group) according to a randomization list. Randomization was concealed, as allocation was generated by a computer program and not known before to the study personnel. Blinding with a placebo-injection in the control group was not possible because subcutaneous injections with mistletoe preparations result in local reactions at the injection site which deblind patients and the physician [26].

Primary outcome parameter was Quality of Life improvement during the 4 visits. aVQ was injected subcutaneously three times a week from postoperative day 7 to week 24 with increasing doses (8 injections 0.02 mg followed by 8 injections 0.2 mg, 8 injections 2mg and 8 injections 20mg and then continued with 20mg to the end of the study). This schedule corresponds to the recommended treatment schedule of the manufacturer.

The 5-fluorouracil prodrug 5-DFUR is approved to treat gastric cancer in Japan and Korea and is used as adjuvant treatment in a dose of 600–900 mg per day, depending on the weight of the patient. Chemotherapy started together with the mistletoe treatment one week after operation.

The QoL (EORTC QLQ-C30 and -STO22 questionnaires), liver function tests, peripheral differential blood count, adverse events and immunological parameters (levels of TNF-alpha, IL-2, CD16^+^/CD56^+^ and CD 19^+^ lymphocytes) were recorded at each visit (baseline, week 8, 16 and 24). The immunological parameters were analyzed in the quality controlled, accredited laboratory Seoul Clinical Laboratories with established methods.

### Medication

AbnobaVISCUM® Q 20 mg is an injectable, endotoxin-free plant extract from the European mistletoe species Viscum album L. for the treatment of malignant tumors, tumor recurrences, and defined precanceroses. AbnobaVISCUM® Q 20 mg (1 ampoule a 1 ml) contains about 8500 ng/ml natural mistletoe lectins. The mistletoe lectin content of the diluted preparations is accordingly. 5-DFUR is used in Korea for adjuvant therapy of gastric cancer in early stages and is an oral prodrug of 5-FU.

### Planned number of cases and statistical analysis

As this was a pilot study, 16 patients per group have been planned. The independent *t*-test and repeated measured ANOVA were used for statistical analysis and *P*-values less than 0.05 (two-sided) were regarded as statistically significant. Statistical analysis was performed using SPSS version 12.0 (Chicago, Illinois, USA).

## Results

A total of 32 patients with stage Ib/II gastric cancer, eligible for adjuvant oral doxifluridine treatment, were enrolled into this clinical trial. Three of the enrolled patients, one from the treatment group and two from the control group, were drop outs because they retrieved the informed consent.

No significant differences were observed in age, sex, height, weight, blood pressure, pulse rate, type of operation (total gastrectomy or distal gastrectomy) and pathologic classifications between the two groups (Table 1). In both groups, the number of male patients was significantly higher. There were 28 patients in stage Ib and only one patient in stage II.

**Table 1:** Demographic and pathologic data (SD= standard deviation; BP= Blood pressure, number of drop out patients in

	**Treatment group**	**Control group**	**p-value**
Stage Ib	15 (1)	16 (2)	-
Stage II	1	0	-
Total gastrectomy	4	3	-
Distal gastrectomy	12 (1)	13 (2)	-
Sex (M:F)	13 (2):3	13 (1):3	-
Age (year, mean ± SD, median)	53.75±10.25, 54	54.87±11.51, 52.5	0.77
BMI (mean ± SD)	23.21±2.09	23.57±1.93	0.63
Systolic BP (mmHg, mean ± SD)	112.88±9.23	108.88±9.84	0.24
Diastolic BP (mmHg, mean ± SD)	70.75±7.69	72.38±8.16	0.57
Pulse rates (mean ± SD)	73.06±12.89	74.94±10.88	0.65

Except 3 QoL parameters (pain, p = 0.038; eating restrictions, p = 0.037; hair loss, p = 0.023) and basophiles (p = 0.0315) baseline of QoL, immunological parameters, hematology and liver function tests were not different between the groups (Tables 2, 3, 4, 5).

**Table 2:** **QoL-Questionnaires: difference between treatment group (aVQ) and control group by independent *t*-test and result of the repeated measured analysis of variance (ANOVA, influence of treatment)**

		**Values at baseline (SD)**		**Difference between aVQ and control**				
		**aVQ**	**Control**	**t**	**t**	**t**	**t**	**F**
				**baseline**	**week 8**	**week 16**	**week 24**	**ANOVA**
Global health status		43.75 (12.73)	38.33 (22.45)	−0.8192	−0.7853	−4.3243**	−2.2335*	7.7133**
Function scales QLQ-C30	Physical Function	60.83 (18.52)	59.11 (18.66)	−0.2577	0.7034	−1.1736	−0.4788	0.2499
	Role Function	61.46 (35.34)	54.44 (30.52)	−0.5925	0.9454	−1.616	−0.5529	0.4646
	Emotional Function	60.94 (21.02)	55.56 (28.64)	−0.5933	0.2221	2.2619*	0.0475	0.0851
	Cognitive Function	75.00 (21.94)	77.78 (15.00)	0.4137	1.209	1.5594	−0.2262	1.0832
	Social Function	58.33 (29.81)	58.89 (35.56)	0.047	−0.684	0.7015	−0.396	0.0107
Symptom scales QLQ-C30	Fatigue	44.44 (17.68)	48.89 (27.47)	0.5317	−0.1426	−0.4653	0.3975	0.0812
	Nausea &Vomiting	17.71 (18.73)	5.56 (13.61)	−2.0761	−0.764	−1.1565	0.102	2.6182
	Pain	51.04 (21.49)	55.56 (33.73)	0.4411	−0.9279	−0.2941	0.6467	0.0258
	Dyspnoea	25.00 (28.54)	33.33 (35.63)	0.7157	0.4233	−1.1535	−0.5658	0.0023
	Insomnia	37.50 (29.50)	53.33 (37.37)	1.3036	−1.2579	0.130	−0.8726	0.0214
	Appetite loss	33.33 (27.22)	37.78 (35.34)	0.3905	0.6096	−0.2297	0.7067	0.4156
	Constipation	31.11 (34.43)	22.22 (27.22)	−0.7845	−0.1521	−0.5357	−0.7574	0.7652
	Diarrhea	22.22 (24.12)	15.56 (24.77)	−0.7467	−0.6185	−2.6407*	−1.0367	3.606
	Financial difficulties	37.50 (34.16)	28.89 (27.79)	−0.772	−0.0922	−1.6535	−0.8739	1.0459
Symptoms QLQ-STO22	Dysphagia	34.03 (22.76)	46.67 (21.50)	1.5901	−0.1442	1.0202	0.4126	1.7228
	Pain	43.75 (26.44)	63.89 (25.13)	2.1742*	0.757	−0.7438	0.3681	1.9013
	Reflux symptom	27.08 (15.70)	22.22 (23.76)	−0.6676	−1.2596	−1.7927	−1.0456	3.1538
	Eating restriction	29.17 (22.57)	46.11 (20.62)	2.1843*	0.2106	0.7307	0.1629	2.3484
	Anxiety	42.36 (23.38)	40.00 (28.73)	−0.25	−1.5867	−0.0319	0.4544	0.2471
Single items QLQ-STO22	Having a dry mouth	39.58 (30.35)	57.78 (36.66)	1.4998	−1.0582	−1.1941	−1.3797	0.1782
	Taste	22.92 (23.47)	40.00 (31.37)	1.708	−0.4383	0.7737	0.6099	1.4529
	Body image	41.67 (28.54)	33.33 (30.86)	−0.7791	−2.3885*	−0.0962	0.00	1.4123
	Hair loss	20.00 (18.26)	83.33 (33.33)	3.4125*	0.5143	0.4672	−1.4018	1.4885

*p<0.05, **p<0.01, SD: standard deviation.

**Table 3:** **Immunological parameters: difference between treatment group (aVQ) and control group by independent*t*-test and result of the repeated measured analysis of variance (ANOVA, influence of treatment)**

	**Values at baseline (SD)**		**Difference between aVQ and control**				
	**aVQ**	**Control**	**t**	**t**	**t**	**t**	**F**
			**baseline**	**week 8**	**week 16**	**week 24**	**ANOVA**
CD 16^+^/CD 56^+^	10.5 (4.16)	9.38 (3.61)	−0.816	−0.3458	−0.452	−0.2512	0.2823
CD 19^+^	21.06 (13.71)	20.25 (8.97)	−0.198	0.2786	0.1885	0.3131	0.0199
TNF-α	2.18 (1.61)	1.6 (0.49)	−1.381	−1.4135	−0.407	−1.6893	2.7911
IL-2	1.04 (0.56)	0.94 (0.44)	−0.58	−1.0269	−1.34	−1.019	2.1181

SD: standard deviation; CD 16^+^/CD 56^+^ : lymphocytes with CD 16^+^/CD 56^+^ (NK-cells), CD 19^+^ : lymphocytes with CD 19^+^, TNF-α: tumor necrosis factor alpha, IL-2: interleukin-2.

**Table 4:** **IL-2 mean values and standard deviation (SD): difference between treatment group (aVQ) and control group of the 4 visits by independent *t*-test**

	**IL-2 concentration in pg/ml (mean, (SD), median)**		***t*-test**	
	**aVQ**	**Control**	**t**	**p**
Baseline	1.04 (0.56) 0.89	0.94 (0.44) 0.85	−0.5804	0.5662
Visit 2	59.08 (219.95) 0.93	0.76 (0.52) 0.56	−1.0269	0.3219
Visit 3	243.46 (702.12) 0.75	0.55 (0.42) 0.42	−1.3399	0.2016
Visit 4	70.84 (267.49) 0.81	0.46 (0.38) 0.75	−1.0190	0.3255

IL-2: interleukin-2.

**Table 5:** **Routine laboratory parameters: mean difference and standard deviation (SD) between treatment group (aVQ) and control group by independent *t*-test and result of the repeated measured analysis of variance (ANOVA, influence of treatment)**

	**Values at baseline (SD)**		**Difference between aVQ and control**				
	**aVQ**	**Control**	**t**	**t**	**t**	**t**	**F**
			**at baseline**	**at week 8**	**at week 16**	**at week 24**	**ANOVA**
WBCx1000/μl	6.21 (2.13)	5.24 (1.17)	−1.595	−2.1951*	−2.0243	−2.1076*	7.614*
Platelets x1000/μl	61.38 (77.07)	72.19 (83.77)	0.38	−0.5491	0.2193	0.3207	0.0442
Neutrophils %	64.65 (8)	63.66 (8.29)	−0.345	−0.7475	1.6094	−0.291	0.0055
Lymphocytes %	21.83 (6.37)	22.94 (7.51)	0.4493	2.1109*	−0.068	1.0294	1.5019
Monocytes %	7.07 (2.91)	7.74 (3.69)	0.5746	0.5052	0.2823	0.8871	0.776
Eosinophils %	6.08 (3.89)	4.91 (2.39)	−1.028	−3.677**	−3.2466**	−2.3029*	10.1286**
Basophiles %	0.37 (0.21)	0.76 (0.63)	2.3308*	0.4129	0.0282	−0.081	2.0996
Protein g/dl	6.16 (0.44)	6.31 (0.37)	1.0894	0.1437	0.3964	1.0308	0.4806
AST U/l+	19.62 (4.26)	21 (8.48)	0.5798	0.4339	1.0689	0.4566	0.6618
ALT U/l^†^	19.44 (9.39)	20.31 (11.27)	0.2386	0.064	1.3147	−0.0086	0.2651
ALK U/l‡	65.75 (16.43)	72.56 (48.02)	0.5369	−2.146*	−3.0221**	−2.8479**	2.6545
Bilirubin mg/dl	0.84 (0.24)	0.79 (0.28)	−0.471	−0.1546	−0.4448	−0.6975	0.2735

+AST: aspartate aminotransferase; ^†^ALT: alanine aminotransferase; ‡ ALK: alkaline phosphatase; *p<0.05 **p<0.01.

Repeated analysis of variance (ANOVA) of the quality of life scales QLQ-C30 and QLQ-STO22 revealed a strong improvement of the “Global Health Status” (p = 0.0098) in the intervention group (Table 2). The Global Health Status is a sum parameter of 2 questions of the QLQ-C30 questionnaire with a broader range (range = 6) than the other 28 questions (range = 3), which allows a more precise judgment of the patients situation regarding the overall health and quality of life status. All other function and symptom scales of the QLQ-C30 and the stomach cancer module QLQ-STO22 did not show a significant effect of the intervention. The analysis of variance for the hematologic variables showed significantly higher WBC counts (p = 0.0101) and eosinophil counts (p = 0.0036) in the intervention group. For the immunologic variables repeated measured ANOVA detected no significant differences in CD16^+^/CD56^+^ and CD 19^+^ lymphocytes, TNF-alpha and IL-2 between control group and intervention group (Table 3). Anyhow, the IL-2 mean values are considerably higher in the treatment group (Table 4) with an extreme coefficient of variation. Applying the non-parametric rank sum test for the IL-2 values a significant difference results (F = 4.4794; p = 0.0433). Also the IL-2 median at visit 3, but not at the other visits, was significantly higher in the intervention group compared to the control group (Table 4, p=0.034, two sided Mann-Withney *U*-test).

Mean alkaline phosphatase (ALK) values were higher in the in the treatment group (visit 2, 3, 4, Table 5). Anyhow, a significant influence of the mistletoe treatment on the ALK values could not be confirmed by ANOVA (F = 2.6545, p = 0.1145). Increase of ALK is a known side-effect of doxifluridine therapy.

The number of adverse events was similar in the control group (n = 96) and the treatment group (n = 92). Except 2 serious adverse events (SAE’s), 1 case of post-operative bleeding and 1 case of an acute infection, both in the treatment group and 1 AE with severe degree (itching at injection site, treatment group) all AE´s were mild or moderate. The SAE’s were judged as not related to the study medication. In the treatment group 26 of the 92 adverse events had at least a possible relationship to the mistletoe treatment. 80% (21 cases) of them were related to reactions at the injection site like local pain, itching, rash or urticaria. The others were 1 case of chest pain, 1 case of myalgia, 1 case of dizziness and 1 case of diarrhea. In the control group 20 cases of diarrhea (21% of the 96 cases) were recorded. The difference in diarrhea (6.7% in the treatment group, 50% in the control group, p = 0.014) was statistically significant. All recorded cases of diarrhea had a mild degree but in 3 patients of the control group the symptoms persisted until the end of the study.

## Discussion

While the effect of treatment with mistletoe preparations on survival of cancer patients is still unclear because adequate studies are lacking, there is an increasing number of studies showing beneficial effects regarding QoL [12,25]. Our controlled, randomized pilot study is in line with these findings, showing that also in patients with gastric cancer during adjuvant oral chemotherapy treatment with a mistletoe extract significantly improved the global health status (p<0.01). Interestingly, except the lower frequency of diarrhea, no specific improvement during mistletoe treatment occurred (Table 2). Possible mechanisms how mistletoe treatment could improve the global health status include immunological effects and elevating body temperature [27], because immunological disturbances and an altered circadian rhythm are factors that may contribute to reduced QoL in cancer patients [28].

White blood cell count and eosinophils increased in the treatment group compared to the control group. These effects have been reported in other studies evaluating immunological effects of mistletoe preparations and mistletoe lectins in healthy probands and are most likely related to a stimulation of GM-CSF, IL-5 and IFN-gamma by mistletoe lectin [15,29]. The increase in eosinophils and WBC might therefore also in our study with cancer patients be related to a stimulation of IL-5 and/or GM-CSF Apart from WBC and eosinophils there were no significant differences in immune parameters between the groups.

IL-2 function seems to contribute considerably to the operation-induced immunosuppression in gastric cancer patients [30] and preoperative treatment with IL-2 had been promising [31,32]. Applying a non-parametric statistical method (rank transformation) the IL-2 values significantly increased in the group of gastric cancer patients receiving mistletoe treatment. This can, however, be explained by outliers. Peak levels were measured 7 times only in the treatment group and not in the control group. Analysis of medians showed significantly higher values at visit 3 in the intervention group. Anyhow, the IL-2 increase might have been missed in ANOVA analysis due to the low number of patients or might have been compromised by the concurrent chemotherapy. To distinguish this effect certainly a much larger number of patients would be necessary. Nevertheless it can be assumed that the increased WBC and eosinophil count and possibly also the strong IL-2 increase in individual patients are a result of the immunomodulatory effect of the mistletoe extract.

The compliance regarding administration of the mistletoe extract was good. There was no drop-out related to the investigated medication. The tolerability and safety of the medication were also good. Mistletoe treatment related local reactions at the injection site did not lead to a discontinuation of treatment. With the exception of local reactions, there were no significant differences concerning number of adverse events or laboratory parameters between the intervention group and the control group. Moreover, in the intervention group, diarrhea was less frequently reported than in the control group (7% versus 50%, p=0.014). Reduced diarrhea has also been reported in a non-interventional trial when abnobaVISCUM was given in parallel to adjuvant chemotherapy in breast cancer patients [33]. Mucosal injury is a relevant side effect of modern antineoplastic therapies caused by cytotoxicity, apoptosis induction and anti-angiogenesis [34] and is typical for FU-based chemotherapies [35]. The beneficial effect of the mistletoe therapy on diarrhea could possibly be explained by its immunomodulating properties which might have inhibited apoptosis in the normal gut mucosa. This needs, however, further investigations.

## Conclusions

In this pilot trial the mistletoe extract was safe, improved the global health status and reduced the rate of diarrhea in gastric cancer patients receiving adjuvant oral chemotherapy.

## Competing interests

JE is working for Abnoba company which produces the mistletoe product used in this trial. RH obtained compensation from Abnoba company for scientific projects but not in relation to this study. The study was performed investigator initiated and financed from Abnoba GmbH and Abnoba Korea co. Ltd.

## Authors contributions

KCK and JHY conducted and coordinated the study and significantly contributed to the study protocol. BSK was the principle investigator of the study and wrote the study protocol. JE monitored the data and checked the analyses. RH reviewed the data and prepared the manuscript. All authors have read and approved the final manuscript.

## Pre-publication history

The pre-publication history for this paper can be accessed here:

http://www.biomedcentral.com/1472-6882/12/172/prepub
